# Lazarus effect in a patient initially empirically treated with osimertinib for *EGFR* L858R mutant non-small cell lung cancer with leptomeningeal disease: a case report

**DOI:** 10.18632/oncotarget.28550

**Published:** 2024-01-16

**Authors:** Shreya Bhatia, Manuel G. Cortez, Spencer Lessans, Wade T. Iams

**Affiliations:** ^1^Vanderbilt-Ingram Cancer Center, Nashville, TN 37232, USA

**Keywords:** *EGFR* mutation, leptomeningeal disease, non-small cell lung cancer, osimertinib

## Abstract

Osimertinib has been shown to be effective for patients with non-small cell lung cancer (NSCLC) with activating *EGFR* mutations, and these patients are at risk for leptomeningeal disease. In this report, we present a patient of East Asian descent whose initial presentation included severe, progressive leptomeningeal carcinomatosis and a small lung mass, with limited tissue available for molecular testing. She responded to empiric, urgent initiation of osimertinib, repeat tissue sampling revealed an *EGFR* L858R mutation, and she has experienced durable disease improvement for 18 months on osimertinib monotherapy.

## INTRODUCTION

Osimertinib is a highly effective third generation epidermal growth factor receptor tyrosine kinase inhibitor (EGFR TKI) that is the current first line standard of care for patients with metastatic non-small cell lung cancer (NSCLC) with *EGFR* activating mutations. Importantly, osimertinib provides anti-tumor efficacy for patients with central nervous system (CNS) involvement, either intracranial metastases or leptomeningeal disease (LMD) [1].

Clinically, the early identification of LMD is difficult, and patients often present with diffuse, clinically debilitating LMD. Furthermore, isolation of sufficient tissue for molecular analysis in patients with LMD is a barrier to allocating patients to optimal therapy. Herein, we present the case of a patient who presented with life threatening LMD with insufficient tissue for molecular analysis, who was urgently started on empiric osimertinib with successful disease improvement for over 18 months to date on osimertinib monotherapy.

## CASE REPORT

A 64-year-old woman with no smoking history and of East Asian descent presented to the emergency department with four months of headaches, one month of right eye vision loss, right lower extremity weakness, and syncope. Upon admission, she was also noted to have a spontaneous pneumothorax with an 18 mm spiculated lesion in her left upper lobe (LUL).

A non-contrast MRI of the brain and spine showed no intracranial abnormalities and no LMD was noted. Cerebrospinal fluid (CSF) cytology raised concern for malignancy with large, atypical cells. Bronchoscopy with biopsy of the LUL lesion revealed NSCLC (adenocarcinoma), and carcinomatous meningitis from NSCLC was suspected given the CSF findings. The lung tissue and CSF cytology were insufficient for molecular analysis. Peripheral blood circulating tumor DNA using a Tempus xF^®^ assay was assessed, and there were no pathogenic mutations identified.

**Figure 1:**
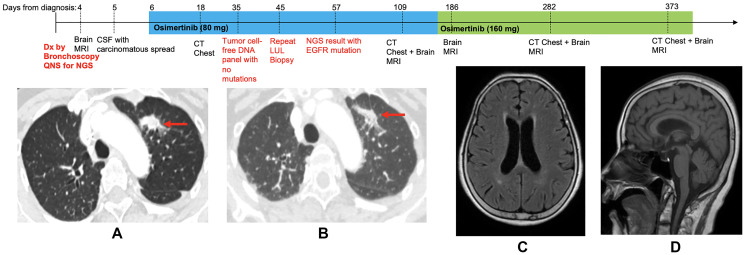
Treatment timeline with chest computed tomography (CT) and brain magnetic resonance images (MRI) on osimertinib. (**A**) Pretreatment CT chest, demonstrating 18 mm spiculated mass in the left upper lobe (LUL). (**B**) CT chest following 12 days of Osimertinib treatment, showing decreased size of LUL mass. (**C**) Pretreatment brain MRI showing no acute abnormalities (axial). (**D**) Pretreatment brain MRI showing no acute abnormalities (sagittal).

Because of the high pre-test probability of this cancer being driven by an *EGFR* activating mutation and the patient’s ongoing clinical decline from carcinomatous meningitis, osimertinib 80 mg daily was empirically started. Within two days headaches improved, and within 2 weeks she had a dramatic improvement in her functional status. Imaging after approximately one month on therapy showed slight improvement in her lung nodule (Figure 1). Repeat bronchoscopy with biopsy of the LUL lesion was completed and Tempus xT^®^ molecular assessment revealed an *EGFR* L858R activating mutation.

The patient required escalated dosing of osimertinib to 160 mg daily for progressive cranial nerve deficits from LMD six months into therapy, and she has since continued osimertinib at 160 mg daily for a total of 18 months to date with ongoing excellent disease control in the lung and CNS.

## DISCUSSION

Leptomeningeal disease is characterized by CNS metastases with spread to the CSF or leptomeninges. In patients with NSCLC with *EGFR* activating mutations, there is an increased occurrence of LMD [2], which occurs in 9% of patients [3].

Osimertinib is an EGFR TKI that has been shown to be safe and efficacious for patients with metastatic *EGFR* mutant NSCLC complicated by LMD [1]. Notably in the BLOOM trial, patients who had previously progressed on other TKI therapies showed clinical improvement in neurological function on osimertinib 160 mg daily [1]. Current data on the use of osimertinib supports its application in previously treated or untreated patients with metastatic *EGFR* mutant NSCLC with LMD.

Our case demonstrates the nuances of decision-making in starting osimertinib in urgent clinical settings. Given our patient’s progressively worsening functional status and spread of disease to her CNS upon presentation, there was a need to begin treatment imminently. Time constraints, financial constraints, and lack of sufficient tissue for analysis ultimately led to the empiric use of osimertinib. Through the urgent initiation of appropriate anti-cancer therapy, she experienced both a life saving improvement in functional status and improvement in her LUL primary tumor one month into treatment.

## CONCLUSION

Overall, this case supports a body of literature noting potentially dramatic clinical benefits of administering appropriate oncogene directed targeted therapy for critically ill patients with NSCLC (Table 1), and in this specific case it is noted that epidemiology can help guide empiric treatment in patients with prohibitive challenges to adequate and timely molecular assessment, an approach that has also been report on two previous occasions (Table 2).

**Table 1:** Lazarus responses with targeted therapies in non-small cell lung cancer

Study	Oncogenic mutation	Clinical status	Treatment	Disease response
Ninomaru 2021 [4] (Kobe, Japan)	MET Exon 14	Leptomeningeal disease	Tepotinib	ECOG PS 3 to 1 PFS 5 months
Facchinetti 2021 [5] (Villejuif, France)	ROS1	Leptomeningeal disease	Lorlatinib	PFS 9 months
Beninato 2020 [6] (Milan, Italy)	ROS1	Respiratory failure	Crizotinib	PFS >2 months
Ahn 2013 [7] (Seoul, South Korea)	ALK	Respiratory failure	Crizotinib	PFS 3 months (2 patients) PFS 8 months (1 patient)
Kerrigan 2016 [8] (Columbus, USA)	ROS1	Respiratory failure	Crizotinib	PFS 16 months
	EGFR	Respiratory failure	Erlotinib	PFS 8 months
Adam 2015 [9] (Leuven, Belgium)	ALK	Respiratory failure	Ceritinib	PFS >1 year
Van Geffen 2013 [10] (Groningen, The Netherlands)	ALK	Bilateral LE paresis and blindness	Crizotinib	PFS 12 months

**Table 2:** Empiric treatment with an EGFR tyrosine kinase inhibitor

Study	Oncogenic mutation	Clinical status	Treatment	Disease response
Bosch-Barrera 2014 [11] (Girona, Spain)	EGFR exon 19 deletion	Respiratory failure	Erlotinib	PFS >6 months
Jeong 2016 [12] (Seoul, South Korea)	EGFR exon 19 deletion	Respiratory failure	Erlotinib	PFS 18 months
